# Dedicator of cytokinesis 8 deficiency and hyperimmunoglobulin E syndrome: A case report

**DOI:** 10.1097/MD.0000000000028807

**Published:** 2022-02-04

**Authors:** Zhaojun Wang, Yanan Zhang, Gang Li, Lingyan Huang, Juan Chen

**Affiliations:** a Department of Pulmonary and Critical care Medicine, General Hospital of Ningxia Medical University, Yinchuan, Ningxia, China; b Department of Critical Care Medicine, General Hospital of Ningxia Medical University, Yinchuan, Ningxia, China; c Central of Medical Laboratory, General Hospital of Ningxia Medical University, Yinchuan, Ningxia, China; d Department of Pathology, General Hospital of Ningxia Medical University, Yinchuan, Ningxia, China.

**Keywords:** dedicator of cytokinesis 8, hyperimmunoglobulin E syndrome, Job syndrome, metagenome next-generation sequencing, pneumonia

## Abstract

**Rationale:**

Hyperimmunoglobulin E syndrome (HIES) is a rare and complex immunoregulatory multisystem disorder characterized by recurrent eczema, skin and sinopulmonary infections, elevated serum immunoglobulin E levels, and eosinophilia. Onset is most likely in childhood, although infrequent adult cases have been reported. Early diagnosis is important. The use of the National Institutes of Health scoring system and the HIES signal transducer and activation of transcription 3 score can standardize the diagnosis of HIES.

**Patient concerns:**

A 19-year-old woman presented with complaints of dry cough, pyrexia, dyspnea, and recurrent pneumonia. She had a history of milk allergy, recurrent eczema, suppurative otitis media, chalazia, and aphthous ulcers. Her parents had a consanguineous marriage.

**Diagnosis:**

HIES; severe pneumonia.

**Interventions:**

Voriconazole (200 mg iv 2 times/d) and flucytosine (1 g orally 4 times/d) for 3 weeks were administered, followed by oral administration of fluconazole for 3 weeks.

**Outcomes:**

The patient experienced near-complete remission of her respiratory symptoms. The patient was followed-up for one and a half years. During the follow-up, the patient presented again with cough and dyspnea and was again admitted to hospital. After being hospitalized for 3 weeks of antibiotic treatment, the patient experienced near-complete relief of her respiratory symptoms.

**Lessons:**

Regardless of patient age, it is important to consider the possibility of HIES when a patient has recurrent eczema, skin and sinopulmonary infections, elevated serum immunoglobulin E levels, and eosinophilia. Early diagnosis and intervention are essential to improve prognosis.

## 1 Introduction

Hyperimmunoglobulin E syndrome (HIES) is a rare multisystem disorder of unknown origin that most frequently arises during childhood. This disorder is characterized by recurrent eczema, skin and sinopulmonary infections, elevated serum immunoglobulin E (IgE) levels, and eosinophilia, and was previously known as Job syndrome.^[1]^ Further research has revealed dominant negative mutations in the signal transducer and activation of transcription 3 (*STAT3*) gene as the etiology of autosomal dominance, leading to this disorder being classified as autosomal dominant HIES (AD-HIES). Subsequently, homozygous and compound heterozygous mutations in the dedicator of the cytokinesis 8 (*DOCK8*) gene were identified in a subset of individuals diagnosed with autosomal recessive HIES (AR-HIES). Each of these genetic etiologies leads to distinct clinical features, yet these 2 disease types have several characteristics in common. More recently, other monogenic disorders of the HIES phenotype have been described; these include mutations in tyrosine kinase 2, phosphoglucomutase 3, serine peptidase inhibitor Kazal type 5, interleukin 6 signal transducer, zinc finger protein 341, caspase recruitment domain family member 11, and Erbb2 interacting protein genes, expanding the genetic heterogeneity of this syndrome.^[2]^

An in-depth literature search revealed that only 60 HIES cases from mainland China have been reported; the mean age at diagnosis was 9 years and the majority was AD-HIES.^[3]^ We here report a 19-year-old woman who presented with classical symptoms of Job syndrome, including recurrent skin infections, eczema, and pulmonary infections. HIES-related gene mutation analysis was performed by next-generation sequencing, and homozygous *DOCK8* mutation was subsequently confirmed.

## 2 Case presentation

Written informed consent was obtained from the patient and her family, and ethics approval was obtained from the ethics committee at the General Hospital at Ningxia Medical University (2021-14).

A 19-year-old woman presented with complaints of dry cough, fever, and shortness of breath for 10 days. The patient's reported fever was initially low-grade, but gradually became high-grade and was accompanied by shortness of breath. The patient was admitted to the local hospital with similar complaints, and a chest computed tomography (CT) scan showed multiple cavities and consolidation. Sputum cultures were positive for methicillin-resistant *Staphylococcus aureus* and *Escherichia coli*. The patient's symptoms were not alleviated following administration of intravenous antibiotics. The patient was admitted to the emergency department of our hospital.

The patient's parents had a consanguineous marriage. The patient was allergic to milk from infancy, and had a history of recurrent eczema and pulmonary infections. Four years earlier, she had recurrent suppurative otitis media, recurrent chalazia, and aphthous ulcers. A routine blood test performed 3 years earlier showed eosinophilia; lymphadenopathy was also found on physical examination. A lymph node biopsy performed at that time revealed eosinophilic infiltration.

**Figure 1:**
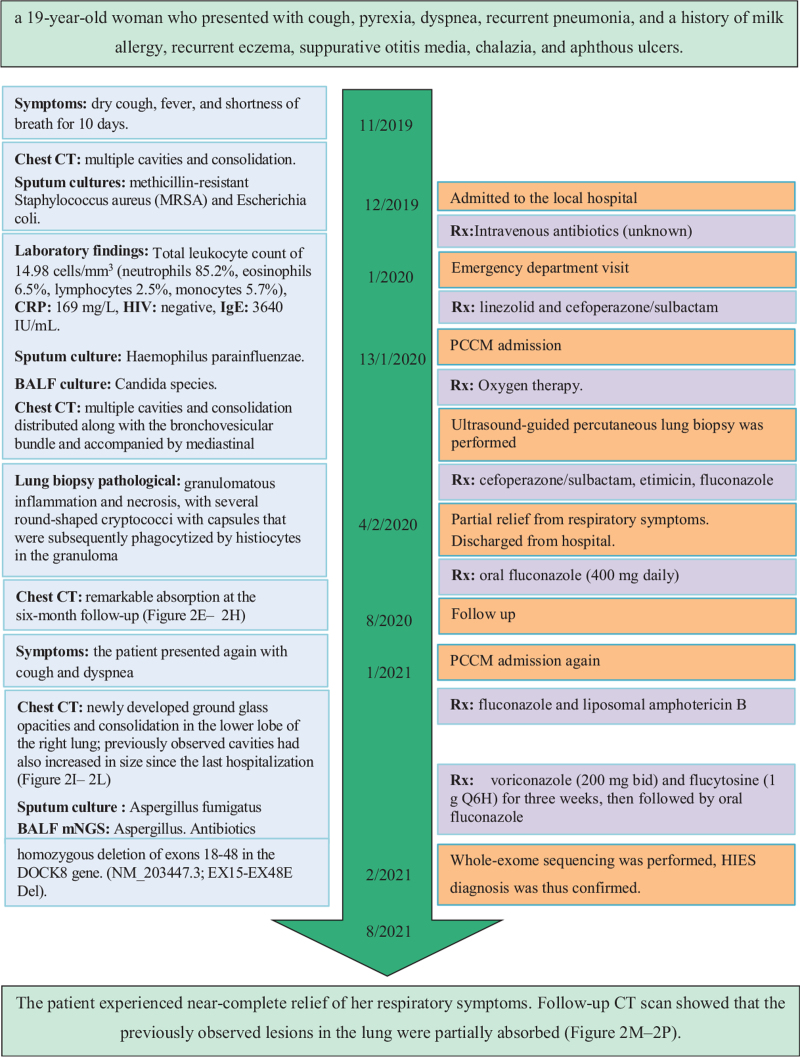
Overview of the patient's clinical presentation and laboratory findings. BALF = bronchoalveolar lavage fluid, CT = computed tomography, CRP = C-reactive protein, DOCK8 = dedicator of cytokinesis 8, HIV = Human Immunodeficiency Virus, IgE = immunoglobulin E, PCCM = pulmonary and critical care medicine.

Laboratory findings showed an elevation of the IgE level to 3640 IU/mL, a hemoglobin level of 7.3 g/dL, a total leukocyte count of 14.98 cells/mm^3^ (neutrophils 85.2%, eosinophils 6.5%, lymphocytes 2.5%, monocytes 5.7%), and a C-reactive protein level of 169 mg/L. The antibody test for the human immunodeficiency virus was negative. Sputum culture revealed the presence of *Haemophilus parainfluenzae*, and bronchoalveolar lavage fluid (BALF) culture revealed the presence of various *Candida* species. Serum cryptococcal antigen was positive. Metagenome next-generation sequencing (mNGS) of the patient's BALF revealed *Klebsiella pneumoniae* and *Cryptococcus neoformans* (Fig. 1).

**Figure 2:**
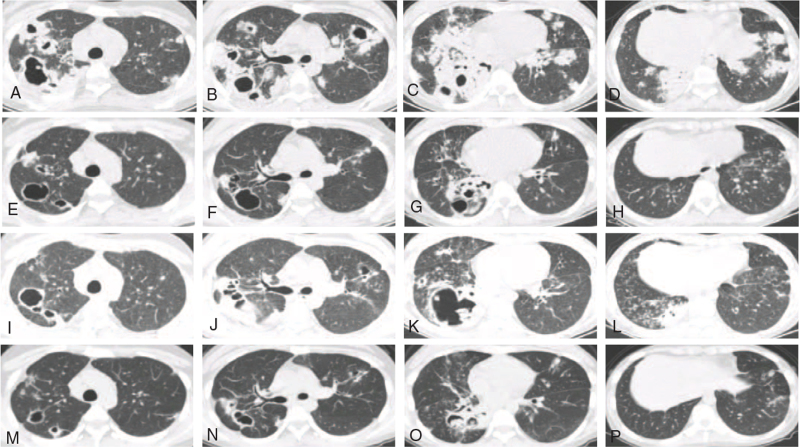
Follow-up chest computed tomography scans. A–D: Onset, E–H: After *C neoformans* target treatment (6 months ago), I–L: Recurrence pulmonary infection, M–P: After *Aspergillus* target treatment (3 months ago).

**Figure 3:**
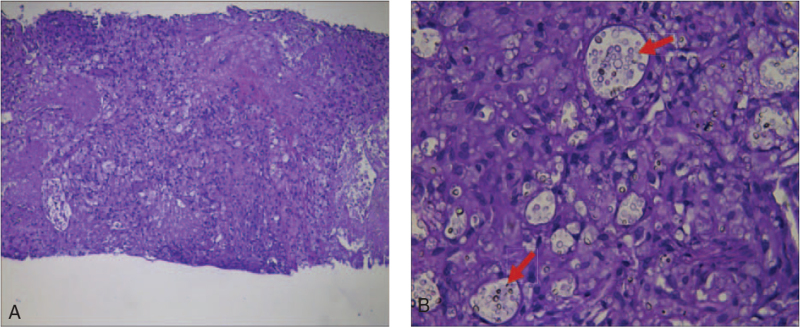
Histological findings of a percutaneous lung puncture biopsy. A: Hematoxylin and eosin staining of the percutaneous lung biopsy showing granulomatous inflammation and necrosis (magnification × 100). B: Hematoxylin and eosin staining of the percutaneous lung biopsy showing granulomatous inflammation and necrosis (magnification × 400).

Chest CT revealed multiple cavities and consolidations distributed along with the bronchovesicular bundle and accompanied by mediastinal adenopathy (Fig. 2A–D); an ultrasound-guided percutaneous lung biopsy was performed before the release of the culture results. Pathological examination showed granulomatous inflammation and necrosis, with several round-shaped cryptococci with capsules that were subsequently phagocytized by histiocytes in the granuloma (Fig. 3). The culture of the patient's lung tissue was positive for *C neoformans*.

Based on the lung tissue culture report and the BALF mNGS, the patient's antibiotics were adjusted to include a 3-week course of fluconazole, cefoperazone/sulbactam, and etimicin. The patient experienced partial relief from her respiratory symptoms during treatment and was discharged from the hospital after completing intravenous antibiotic therapy. The patient began a regimen of oral fluconazole intake (400 mg daily) after hospital discharge and was regularly followed up for 6 months after discharge. During this time, her symptoms significantly improved, and the patient's chest CT scan showed remarkable absorption at the 6-month follow-up (Fig. 2E–H).

One year later, the patient presented again with cough and dyspnea and was again admitted to our hospital. The high-resolution CT scan showed newly developed ground glass opacities and consolidation in the lower lobe of the right lung; previously observed cavities had also increased in size since the last hospitalization (Fig. 2I–L). Sputum culture was positive for *Aspergillus fumigatus*, and the serum cryptococcal antigen was again positive. Serum galactomannan testing was negative; bronchoscopy was performed, and the BALF galactomannan testing was also negative. Subsequent BALF mNGS revealed *Aspergillus*. Antibiotics were adjusted to include voriconazole (200 mg bid) and flucytosine (1 g Q6H) and were followed by oral fluconazole administration (Fig. 1). The patient experienced near-complete relief of her respiratory symptoms after being hospitalized for 3 weeks of antibiotic treatment, and a follow-up CT scan showed that the previously observed lesions in the lung were partially absorbed (Fig. 2M–P).

HIES was suspected in this case based on the combination of recurrent pulmonary infections, high levels of serum IgE, and eosinophilia. The National Institutes of Health (NIH)-HIES score was 46, and genetic assessment was performed on the patient and her immediate family members (parents and elder sister). Whole-exome sequencing was performed according to standard protocols. Genetic testing revealed a large homozygous deletion of exons 18 to 48 in the *DOCK8* gene (NM_203447.3; EX15-EX48E Del). HIES diagnosis was, thus, confirmed.

Hematopoietic stem cell transplantation (HSCT) is the recommended curative option for this patient, but the patient refused HSCT for financial reasons. The patient was followed-up for one and a half years. The patient experienced complete relief of her respiratory symptoms, but is still immunocompromised and very vulnerable to infection.

## 3 Discussion and conclusion

David et al first described the “Job syndrome” in 1966. They reported 2 patients with eczema and recurrent staphylococcal abscesses since birth; the presence of skin boils led these researchers to call this condition “Job syndrome” in reference to the boil- and sore-marked skin of the prophet Job described in the Bible.^[4]^ Elevated IgE levels have since been recognized as a clinical characteristic of this syndrome. Researchers discovered an association between this disease with hyper-IgE.^[5]^ In 1999, Grimbacher et al reported that HIES had an autosomal dominant inheritance. Since that time, HIES has been increasingly recognized as a multisystem immune deficiency involving skin, lung, skeletal, connective tissue, and dental abnormalities.^[6]^ In 2004, Liu et al^[7]^ reported the cases of 13 patients from 6 consanguineous families suffering from HIES and proposed the classification of HIES into 2 subgroups, namely AD-HIES and AR-HIES. Mutations in the *STAT3*, *DOCK8*, and tyrosine kinase 2 genes – along with numerous other gene deficiencies – have been subsequently reported in HIES.^[5]^

HIES is a primary multisystem immunodeficiency disease, and its primary cause remains unknown. It is thought that the gene(s) responsible for HIES influence the many systems involved in HIES manifestations, including not only the regulation of IgE but also abnormalities of the immune system, skeleton, connective tissues, and vasculature. We searched the main HIES-associated genes on the GeneCards website (https://www.genecards.org) using the following keywords and terms: “Hyperimmunoglobulin-E syndrome”; “HIES”; “Job syndrome”; “Buckley syndrome”; “Job–Buckley syndrome”; and “Hyperimmunoglobulin E-recurrent infection syndrome”. A detailed description of the related genes is shown below (Table 1).

**Table 1:** Major genetic mutations in the hyperimmunoglobulin E syndrome gene.

Symbol	Description	Inheritance
STAT3	Signal transducer and activator of transcription 3	AD
CARD11	Caspase recruitment domain family member 11	AD
PGM3	Phosphoglucomutase 3	AR
DOCK8	Dedicator of cytokinesis 8	AR
IL6ST	Interleukin 6 signal transducer	AR
TYK2	Tyrosine kinase 2	AR
ZNF341	Zinc finger protein 341	AR

AD = autosomal dominant, AR = autosomal recessive.

The diagnosis of HIES is dependent on the presence of multisystemic symptoms and signs, including those involving the skin, bones, teeth, and lungs; immunity; and infectious susceptibility. In 1999, the NIH proposed the use of a clinical and laboratory scoring system to assist in HIES diagnosis; per this NIH scoring system, patients with scores of more than 40 are highly likely to have AD-HIES, particularly when recurrent staphylococcal pneumonia or cutaneous abscesses complicate chronic eczema are present. NIH-HIES scores in the range of 20 to 40 represent an intermediate probability of AD-HIES, or another genetic defect associated with HIES.^[7]^ Osteopenia or bone fractures are often present in AD-HIES, and *STAT3* mutation is the major genotype of AD-HIES. Deng et al^[8]^ proposed a series of diagnostic guidelines for STAT3-deficient HIES and use of the HIES STAT3 score, with a total number of scaled points greater than 30 predicting the presence of a *STAT3* mutation.

DOCK8 is a member of the DOCK180 family of guanine nucleotide exchange factors. *DOCK8* mutations occur in AR-HIES. In 2009, Engelhardt et al^[9]^ demonstrated an AR-HIES genomic locus and proposed mutations in the *DOCK8* gene as the major defect in AR-HIES.^[10–14]^ DOCK8 deficiency is associated with impaired activation of CD4^+^ T and CD8^+^ T cells. Patients with DOCK8 deficiency were diagnosed as AR-HIES, involving a high mortality. Subsequent research has revealed that HSCT is a curative therapy. Al-Herz et al^[15]^ suggested that HSCT should be considered as early as possible before the development of a significant organ damage or virus-driven malignancies. HSCT is the recommended curative option for DOCK8 deficiency.

In researching this case, we conducted a thorough search of the HIES-associated literature in PubMed databases using the following search term combinations: “Hyper IgE syndrome” and “DOCK8”, “Job syndrome” and “DOCK8”, “HIES” and “DOCK8”, and “Buckley syndrome” and “DOCK8”. We found 35 publications about HIES resulting from a *DOCK8* mutation. Most patients with a *DOCK8* mutation have symptoms similar to those resulting from STAT3 deficiency, such as eczema, recurrent pneumonia, and elevated serum IgE levels. However, connective tissue abnormalities and skeletal abnormalities are more frequent when a *DOCK8* mutation is present. STAT3-HIES patients more frequently present with retained primary teeth, minimal trauma fractures, scoliosis, and characteristic facial appearance.^[16]^

In summary, HIES is a rare primary immunodeficiency disorder with an unknown cause. Early diagnosis and intervention are essential to improve prognosis. Furthermore, the identification of specific genetic mutations has proven beneficial in detecting the immunological abnormalities and complex manifestations observed in HIES. By reporting this rare case, we highlight the clinical features of HIES and share our experience in the treatment and follow-up of a teenage patient with this disease, with the hope that our clinical experience may prove helpful in the future.

## Acknowledgments

We thank Dr Xin Su, MD, PhD, Department of Respiratory and Critical Care Medicine, Jinling Hospital, Medical School of Nanjing University. During the treatment of this patient, Dr Xin Su provided the suspected diagnosis of HIES.

## Author contributions

ZJW, YNZ, GL, LYH, and JC participated in the care of the patient and contributed to the design of the case report. ZJW drafted the manuscript. JC made major contributions in editing the manuscript. All authors read and approved the final manuscript.

**Resources:** Yanan Zhang, Gang Li, Lingyan Huang.

**Writing – review & editing:** Zhaojun Wang, Juan Chen.
